# The role of ureteric indocyanine green fluorescence in colorectal surgery: a retrospective cohort study

**DOI:** 10.1007/s10151-024-02955-x

**Published:** 2024-07-10

**Authors:** P. Rogers, J. Dourado, A. Wignakumar, B. Weiss, P. Aeshbacher, Z. Garoufalia, V. Strassmann, S. Emile, P. Strzempek, S. Wexner

**Affiliations:** 1 Ellen Leifer Shulman and Steven Shulman Digestive Disease Center, Cleveland Clinic Florida, 2950 Cleveland Clinic Blvd, Weston, FL 33331 USA; 2 Department of Visceral Surgery and Medicine, Inselspital, Bern University Hospital, University of Bern, Bern, Switzerland; 3 Colorectal Surgery Unit, General Surgery Department, Mansoura University Hospitals, Mansoura, Egypt; 4 Ross University School of Medicine, Miramar, USA

**Keywords:** Indocyanine green, Fluorescence imaging, Identification, Ureters, Colorectal surgery

## Abstract

**Background:**

Ureteric injury (UI) is an infrequent but serious complication of colorectal surgery. Prophylactic ureteric stenting is employed to avoid UI, yet its efficacy remains debated. Intraoperative indocyanine green fluorescence imaging (ICG-FI) has been used to facilitate ureter detection. This study aimed to investigate the role of ICG-FI in identification of ureters during colorectal surgery and its impact on the incidence of UI.

**Methods:**

A retrospective cohort study involving 556 consecutive patients who underwent colorectal surgery between 2018 and 2023 assessed the utility of routine prophylactic ureteric stenting with adjunctive ICG-FI. Patients with ICG-FI were compared to those without ICG-FI. Demographic data, operative details, and postoperative morbidity were analyzed. Statistical analysis included univariable regression.

**Results:**

Ureteric ICG-FI was used in 312 (56.1%) patients, whereas 43.9% were controls. Both groups were comparable in terms of demographics except for a higher prevalence of prior abdominal surgeries in the ICG-FI group. Although intraoperative visualization was significantly higher in the ICG-FI group (95.3% vs 89.1%; *p* = 0.011), the incidence of UI was similar between groups (0.3% vs 0.8%; *p* = 0.585). Postoperative complications were similar between the two groups. Median stent insertion time was longer in the ICG-FI group (32 vs 25 min; *p* = 0.001).

**Conclusion:**

Ureteric ICG-FI improved intraoperative visualization of the ureters but was not associated with a reduced UI rate. Median stent insertion time increased with use of ureteric ICG-FI, but total operative time did not. Despite its limitations, this study is the largest of its kind suggesting that ureteric ICG-FI may be a valuable adjunct to facilitate  ureteric visualization during colorectal surgery.

## Introduction

Ureteric injury (UI) is a devastating complication in 0.2–7.6% of colorectal operations, with modern large cohorts finding injury rates approximately 0.6% [1–4]. The mechanisms of intraoperative UI include laceration, ligation, crush, division, or devascularization [4]. While prophylactic ureteric stenting has been employed in colorectal surgery, the rates of UI are similar among stented and unstented patients. Additionally, stenting is not without stent-related complications [3, 5, 6]. However, the main virtue of stenting is it enhances the intraoperative recognition of UI once it has occurred [4, 7, 8], which improves outcomes when timely repair is undertaken [4, 9].

Since its approval by the US Food and Drug Administration (FDA) in 1956, indocyanine green fluorescence imaging (ICG-FI) has been used as a safe and effective option for medical diagnosis and enhanced visualization of anatomic structures. Its use in colorectal surgery, particularly in bowel perfusion assessment and lymph node mapping, has been widely adopted [10, 11]. The use of ICG-FI along with ureteric stents has not been extensively assessed in the published literature, but the current published studies show promise in real-time visualization of the ureter [12–15]. Owing to the paucity of studies on the role of ICG-FI in ureter detection, we conducted this retrospective study with the hypothesis that using real-time ICG-FI as an adjunct to ureteric stenting may serve to facilitate the detection of and/or minimize the incidence of UI. Therefore, this study aimed to assess the efficacy of routine prophylactic ureteric stenting with adjunctive injection of ICG-FI in the identification of the ureters, and to determine whether intraoperative and postoperative complications were altered with the use of ureteric ICG-FI.

## Methods

### Study design and setting

This retrospective cohort study was undertaken at a tertiary referral center. We reviewed the records of all consecutive patients who had undergone colorectal surgery between 2018 and 2023 by a single surgeon in whom ureteric stents were utilized. Patients operated on from October 2020 forward had adjunctive ICG-FI injection into the ureters. Our comparison was between two groups: ureteric stenting with ICG-FI and without.

### Stenting, ICG-FI, and ureteric visualization

Bilateral ureteric stenting was performed by attending urologists for all cases. Cystoscopic examination of the bladder was undertaken with subsequent ureteric stent insertion. Ten milliliters of sterile water was mixed with the ICG-FI compound, then 5 ml was injected into each ureter via the catheter. If required as a result of inadequate visualization, ICG-FI was reinjected into the ureters. For the purpose of this study, ureteric visualization was confirmed when it was explicitly stated in the operative report that ureters were visualized.

### Data

Demographic data including age, sex, and comorbidities were analyzed. Operative details, including ureteric injury and operative time, were recorded. Additionally, postoperative morbidity was compared between the two groups.

### Primary and secondary outcomes

The primary outcome was the incidence of UI as confirmed from the operative report, and secondary outcomes included intraoperative time, ureteric visualization, and postoperative morbidity.

### Statistical analysis

Statistical analysis was performed using EZR (version 1.55) and R (version 4.1.2) [16]. Categorical data are expressed as absolute numbers and percentages, with analysis performed using Fisher’s exact or chi-square test. Continuous variables are expressed as mean and standard deviation and, alternatively, median and interquartile range (IQR) when not normally distributed. Further analyses were performed using the Student *t* test or Mann–Whitney test, as appropriate. To identify which factors were associated with ureteric injury, a univariable association analysis was performed.

### Ethics approval

The Institutional Review Board (IRB) of Cleveland Clinic approved this study (IRB number 23-521).

## Results

### Demographics

In total, 556 patients were included in the study, of whom 287 (51.7%) were male. The mean age was 58.3 (SD 16.8) years and mean body mass index (BMI) was 26 (SD 5.9) kg/m^2^. A total of 312 (56.1%) patients underwent ureteric stenting with ICG-FI, whereas 244 (43.9%) underwent stenting only. Malignancy (211; 37.9%) was the most common indication for surgery, followed by diverticular disease (132; 23.7%), and inflammatory bowel disease (122; 21.9%) (Table 1). These demographic data differentiating between ICG and no ICG with further group detail are shown in Table 2. The only significant demographic difference between the groups was a higher number of previous abdominal surgeries within the ICG-FI group (*p* = 0.003). As there was no patient selection, the authors can see no obvious reason for this significance.

**Table 1:** Demographics of the cohort studied

Factor	Subgroup	
Total		556
Sex (%)	Female	268 (48.2)
	Male	287 (51.7)
Mean age at operation (SD)		58.3 (16.8)
BMI (SD)		26 (5.9)
Surgical indication (%)	Diverticular disease	132 (23.7)
	Inflammatory bowel disease	122 (21.9)
	Malignancy	211 (37.9)
	Other	91 (16.3)

*BMI* body mass index, *SD* standard deviation

**Table 2:** Detailed demographic data with group comparison between no ICG and ICG

Factor	Subgroup	Stenting	ICG stenting	*p* value
Total		244	312	
Sex (%)	Female	121 (50.0)	147 (46.9)	0.494
	Male	122 (50.0)	165 (53.1)	
Mean age at operation (SD)		59.00 (20.75)	61.00 (19.5)	0.377
Mean BMI (SD)		25.45 (11.84)	25.44 (13)	0.953
Surgical indication (%)	Diverticular disease	49 (20.1)	83 (26.6)	0.066
	Inflammatory bowel disease	65 (26.6)	57 (18.3)	
	Malignancy	93 (38.1)	118 (37.8)	
	Other	37 (15.2)	54 (17.3)	
Number of prior abdominal operations (%)	0	106 (43.4)	142 (45.5)	0.003*
	1	65 (26.6)	78 (25.0)	
	2	19 (7.8)	47 (15.1)	
	> 2	38 (15.6)	40 (12.8)	
	Unknown	16 (6.6)	5 (1.6)	
Prior abdominopelvic sepsis (%)	No	206 (84.8)	245 (79.5)	0.120
	Yes	37 (15.2)	63 (20.5)	
Prior radiation (%)	No	204 (83.6)	267 (85.6)	0.554
	Yes	40 (16.4)	45 (14.4)	
Type of surgery at commencement of operation (%)	Laparoscopic	240 (98.4)	306 (98.1)	1
	Open	4 (1.6)	6 (1.9)	
	Open conversion	2	2	

*BMI* body mass index, *SD* standard deviation

*Indicates statistical significance

### Surgical outcomes

In total, there were three (0.005%) documented UIs, one in the ICG group and two in the no ICG group (*p* = 0.585); all were left ureteric injuries. When stratified by sidedness of surgery, two UIs occurred during left-sided surgery and one during a total colectomy. The rate of ureteric visualization was significantly higher in the ICG-FI and stenting group (95.3% vs 89.1% *p* = 0.011). The median stent insertion time was significantly higher in the ICG-FI group (32 vs 25 min; *p* = 0.001). There was no significant difference in terms of complications between the two groups. Intra- and postoperative factors are summarized in Table 3.

**Table 3:** Intraoperative and postoperative outcomes: no ICG-FI vs ICG-FI

Factor	Stenting (*n* = 244)	ICG stenting (*n* = 312)	*p* value
Ureteric injury (%)	2 (0.8)	1 (0.3)	0.585
Ureteric visualization	205 (89.1)	285 (95.3)	0.011*
Median stent insertion time (range)	25 (2–104)	32 (2–65)	< 0.001*
Median total surgical time (range)	260.00 (60–664)	272 (90–960)	0.149
Urinary retention (%)	37 (10.2)	35 (9.0)	0.164
Acute kidney injury (%)	17 (7)	25 (7.7)	1
Hematuria (%)	17 (7)	25 (8.1)	0.059
Hydronephrosis (%)	5 (2.1)	9 (2.6)	1
UTI (%)	7 (2.9)	4 (1.3)	0.340

*ICG-FI* indocyanine green fluorescence imaging, *UTI* urinary tract infection

*Indicates statistical significance

### Subanalysis of ureteric injury

Univariate analysis was undertaken to determine factors associated with UI regardless of ICG-FI status (Crohn’s disease, 66.7% vs 10.8%; *p* = 0.034, higher mean BMI, 31 vs 25.4; *p* = 0.04, prior abdominal sepsis, 100% vs 17.7%; *p* = 0.006, and a longer total median operative time, 415 vs 267 min; *p* = 0.023) (Table 4).

**Table 4:** Univariable analysis of ureteric injury

Factor	Group	No ureteric injury	Ureteric injury	*p* value
*n*		553	3	
Sex (%)	Female	266 (48.2)	2 (66.7)	0.612
	Male	286 (51.8)	1 (33.3)	
Mean age at operation (SD)		60.00 (20.7)	57.00 (5.7)	0.609
Mean BMI (SD)		25.40 (13)	31.00 (0.25)	0.040*
Crohn’s disease (%)		60 (10.8)	2 (66.7)	0.034*
UC (%)		66 (11.9)	0 (0.0)	1
Laparoscopic converted to open (%)		4 (0.7)	0 (0.0)	0.063
ICG-FI inserted to stent (%)		311 (56.2)	1 (33.3)	0.585
Index operation performed (%)	Anterior resection	67 (12.1)	0 (0.0)	0.791
	APR	53 (9.6)	1 (33.3)	
	Left hemicolectomy	13 (2.4)	0 (0.0)	
	Right hemicolectomy	96 (17.4)	0 (0.0)	
	Sigmoid resection	109 (19.7)	1 (33.3)	
	Total colectomy	30 (5.4)	1 (0.0)	
	Total proctocolectomy	76 (13.7)	0 (0.0)	
	Other (including subtotal colectomy, segmental colectomy, and unspecified)	109 (19.7)	0 (0.0)	
Number of prior abdominopelvic operations (%)	0	248 (44.8)	0 (0.0)	0.291
	1	141 (25.5)	2 (66.7)	
	2	66 (11.9)	0 (0.0)	
	> 2	77 (13.9)	1 (33.3)	
	Unknown	21 (3.8)	0 (0.0)	
Prior abdominopelvic sepsis (%)		97 (17.7)	3 (100.0)	0.006*
Prior radiation (%)		84 (15.2)	1 (33.3)	0.393
Surgical indication (%)	Diverticular disease	131 (23.7)	1 (33.3)	0.180
	Inflammatory bowel disease	120 (21.7)	2 (66.7)	
	Malignancy	211 (38.2)	0 (0.0)	
	Other	91 (16.5)	0 (0.0)	
Type of surgery at commencement of operation (%)	Laparoscopic	544 (98.4)	2 (66.7)	0.053
	Open	9 (1.6)	1 (33.3)	
Ureters visualized (%)		487 (92.6)	3 (100.0)	1
Median stent insertion time (IQR)		29.00 (71)	40.00 (41)	0.4
Median surgical time (IQR)		267.00 (899)	415.00 (5)	0.023*

*IQR* interquartile range, *SD* standard deviation, *BMI* body mass index, *APR* abdominoperineal resection. *UC* ulcerative colitis

*Indicates statistical significance

### Ureteric injury management

Case 1: Open abdominoperineal resection (APR) pouch excision with no ICG-FI for Crohn’s disease. Hemostatic sutures were invertedly placed around the ureter. This problem was immediately recognized, the urology team inspected the ureter and was satisfied that no damage had occurred.

Case 2: Laparoscopic sigmoid resection with ICG-FI for diverticular disease. Partial transection of the ureter occurred. The urology team performed a primary anastomosis over the ureteric stent. No further complication occurred.

Case 3: Laparoscopic total colectomy with no ICG-FI for Crohn’s disease. Complete transection of the ureter occurred. The urology team performed a primary ureteric anastomosis over a stent. No further complication occurred.

### Subanalysis of ureteric visualization

**Fig. 1:**
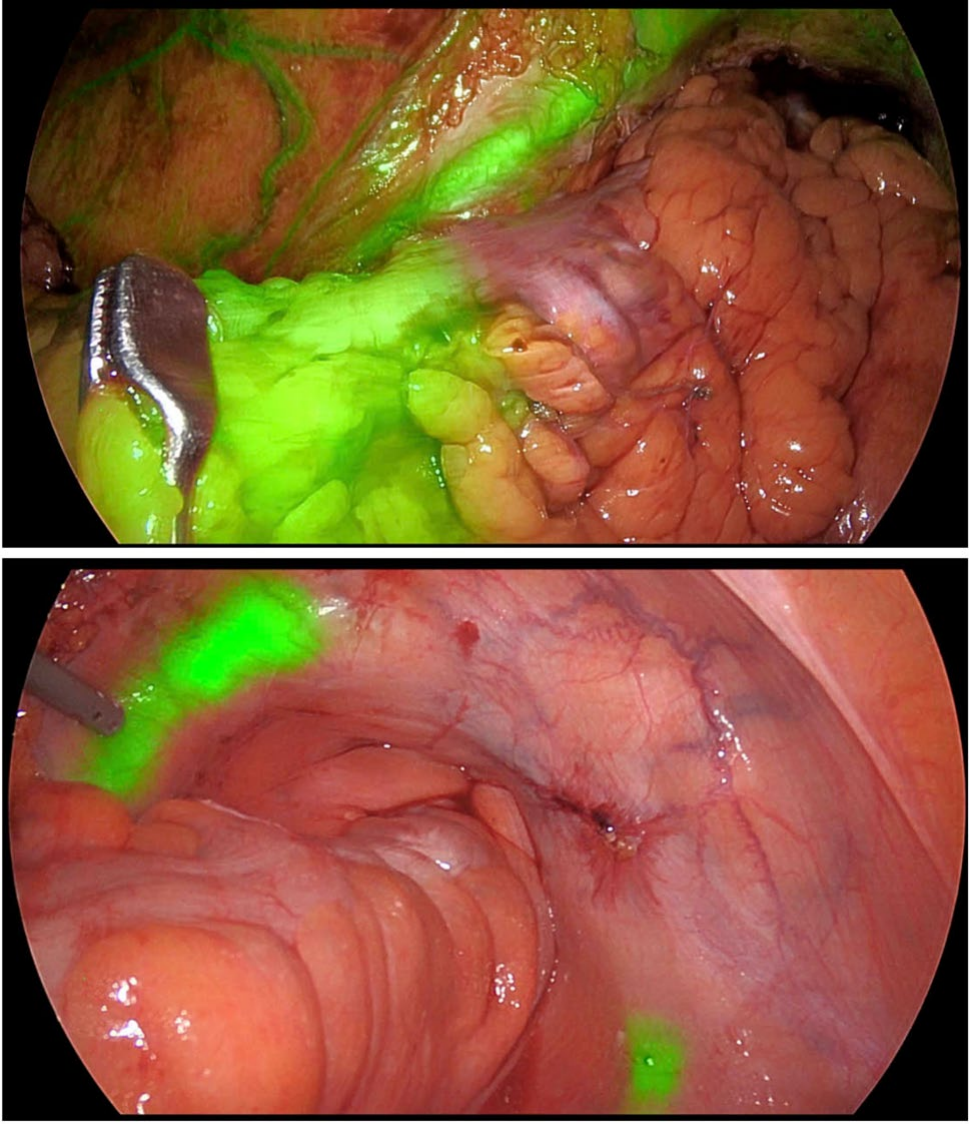
Intraoperative view of the ureter with indocyanine green

To analyze the factors associated with ureteric visualization, we performed a univariate analysis. Only ureteric ICG-FI was significantly associated with ureteric visualization (*p* = 0.007) (Table 5). Multivariate analysis was not performed on this outcome because of low numbers. Figure 1 demonstrates the intraoperative view of ureteric ICG-FI.

**Table 5:** Univariable analysis of ureteric visualization

Factor	Group	Ureters not visualized	Ureters visualized	*p* value
*n*		39	490	
Age at operation (SD)		58.00 (15.5)	61.00 (20.75)	0.205
BMI (SD)		25.40 (4.07)	25.50 (13)	0.223
Sex (%)	Female	18 (46.2)	236 (48.3)	0.868
	Male	21 (53.8)	253 (51.7)	
ICG-FI inserted to stent		14 (35.9)	286 (58.4)	0.007*
Index operation performed (%)	Anterior resection	2 (5.1)	64 (13.1)	
	APR	5 (12.8)	48 (9.8)	0.160
	Left hemicolectomy	0 (0.0)	13 (2.7)	0.941
	Right hemicolectomy	5 (12.8)	85 (17.3)	0.457
	Sigmoid resection	4 (10.3)	102 (20.8)	0.795
	Total colectomy	3 (7.7)	27 (5.5)	0.177
	Total proctocolectomy	4 (10.3)	66 (13.5)	0.457
	Other	16 (41.0)	85 (17.3)	
Prior abdominopelvic sepsis (%)		10 (25.6)	82 (16.7)	0.353
Redo or second stage operation (%)		10 (25.6)	71 (14.5)	0.229
Prior radiation (%)		6 (15.4)	75 (15.3)	1
Surgical indication (%)	Diverticular disease	5 (12.8)	124 (25.3)	0.246
	Inflammatory bowel disease	11 (28.2)	98 (20.0)	
	Malignancy	15 (38.5)	188 (38.4)	
	Other	8 (20.5)	80 (16.3)	

*APR* abdominoperineal resection, *BMI* body mass index, *SD* standard deviation, *ICG-FI* indocyanine green fluorescence imaging

*Indicates statistical significance

## Discussion

The results of this large cohort study demonstrated that ureteric stenting coupled with ICG injection did not significantly reduce the incidence of ureteric injury, compared to only stenting. We did, however, show that intraoperative visualization of the ureters was significantly higher in the ICG group, yet it was accompanied by a modest increase in stenting time. Additionally, subgroup analysis demonstrated that prior abdominopelvic sepsis significantly increased the likelihood of ureteric injury, regardless of ICG use. It must be noted that, with such a low ureteric injury rate, strong conclusions regarding these risk factors are challenging.

The use of ureteric stenting in colorectal surgery has been debated for quite some time. There is conjecture regarding routine use, selected use, or abandonment of stenting all together [2–8]. There has been no large study to date that has clearly demonstrated the benefit in ureteric stenting in relation to ureteric injury prevention. Given the exceedingly low rate of ureteric injury in colorectal surgery, approximately 0.6% [1, 2, 17], as well as the selective stenting in the more challenging cases, it is difficult to obtain robust data on the utility of stenting in prevention of ureteric injury. A meta-analysis of 98,507 patients by Hird et al. [3] in 2020 showed that ureteric stenting was not associated with a decreased likelihood of ureteric injury. Conversely, another meta-analysis of 869,603 patients by Croghan et al. [5] demonstrated a higher likelihood of ureteric injury in stented patients, thought to be a result of selection bias as the more complex patients were stented. The rate of intraoperative recognition of ureteric injury in those who are stented tends to be higher [4, 7, 8], with early repair generally leading to better outcomes [4, 9]. Interestingly, a study by Alexandre et al. [18] showed that despite ureteric stents shifting injury towards intraoperative recognition, this did not offset the healthcare cost of the initial stent insertion.

ICG injection, as an adjunct to ureteric stenting, allows for the direct, real-time visualization of the ureters without the tactile feedback provided by stents alone. Objective visualization of the ureter should lead to a reduction in inadvertent ureteric injury. Our study could not demonstrate that ICG injection with stenting decreased ureteric injury, probably because of the small number of events recorded. However, our study did show enhanced ureteric visualization with ICG stenting. There is some description of ICG identification of ureters in other surgical specialties [19–21], but few within colorectal surgery. Satish et al. [12] in 2021 demonstrated excellent intraoperative visualization of ureters with ureteric ICG injection in two colorectal surgery patients, none of whom had a ureteric injury. Yeung et al. [13] described a novel technique of intravenous methylene blue administration in colorectal surgery patients, with subsequent examination of the ureters with specialized cameras. Of 11 ureters examined, 10 were accurately visualized, and in one case demonstrated the ureter more medial than initially thought by the surgeon. In 2020, White et al. [14] described intraurethral injection of ICG in 15 patients who underwent robotic colorectal surgery, with immediate removal of the stent. This allowed for the successful identification of the ureter in 94% of their patients. Real-time visualization of the ureter without the need for tactile feedback seems to be the main utility of ICG stenting; if this can lead to improved intraoperative recognition of the ureter and ureteric injuries, patient outcomes should logically improve. Additionally, this vivid visualization of the ureteric location can be a very useful teaching tool for trainee surgeons.

Acute kidney injury, urinary tract infection, increased length of stay, and hematuria have all been associated with ureteric stenting [2–8]. Conversely, in Hird’s [3] analysis of 98,507 patients, none of these were significant compared to no stenting. In our review, there was no increase in complication rate with the use of ICG within stents. Looking to the future, the ability to achieve ureteric visualization without the need for stenting may indeed mitigate lost time, increased cost, and risk of stent complications. Mahalingam et al. [22], in a study of five pigs, demonstrated that systemic injection of UreterGlow™ allowed visualization of ureters for more than 2 h. Although experimental, this shows promise for ureteric visualization without the necessity of stent insertion.

In subanalysis of our cohort, independent of ICG use, we found that Crohn’s disease, a higher BMI, prior abdominal sepsis, and a longer total median operative time were significantly associated with ureteric injury. These factors come as no surprise given all predispose to a technically more difficult operation. Additionally, as all ureteric injuries were intraoperatively managed, operative time logically increases. Understandably, it is quite difficult to objectively quantify the utility of ICG stenting in a scarred operative field, like a radiated pelvis, following a leak, or an abdomen with a large phlegmon/abscess. Again, it is important to note that, as a result of the low number of ureteric injuries, strong statements regarding risk factors in this cohort are challenging.

This study is the largest of its kind investigating the use of ICG within ureteric stents in colorectal surgery. Despite this attribute, the study had some limitations. Firstly, it is retrospective in nature which has inherent data flaws and selection bias. Additionally, it was a single-center, single-surgeon study of consecutive patients during two sequential time periods. Finally, the primary outcome of ureteric injury was a rare event, meaning statistical significance was hard to achieve. Further larger cohort studies are required to answer the question on the role of ICG with ureteric stenting in colorectal surgery.

## Conclusion

Our study, despite not demonstrating a reduction in ureteric injury with the use of ureteric ICG-FI, showed a significant improvement in ureteric visualization. Conversely, ICG-FI use did confer an increase stent insertion time but without increasing total operative time. Looking to the future, ICG-FI could be a useful adjunct for the colorectal surgeon to aid in real-time ureteric visualization.

## Data Availability

Upon reasonable request to first author.
