# Correction: The role of ureteric indocyanine green fluorescence in colorectal surgery: a retrospective cohort study

**DOI:** 10.1007/s10151-025-03112-8

**Published:** 2025-02-08

**Authors:** P. Rogers, J. Dourado, A. Wignakumar, B. Weiss, P. Aeschbacher, Z. Garoufalia, V. Strassmann, S. Emile, P. Strzempek, S. Wexner

**Affiliations:** 1 Ellen Leifer Shulman and Steven Shulman Digestive Disease Center, Cleveland Clinic Florida, 2950 Cleveland Clinic Blvd, Weston, FL 33331 USA; 2 Department of Visceral Surgery and Medicine, Inselspital, Bern University Hospital, University of Bern, Bern, Switzerland; 3 Colorectal Surgery Unit, General Surgery Department, Mansoura University Hospitals, Mansoura, Egypt; 4 Ross University School of Medicine, Miramar, USA

**Correction: Techniques in Coloproctology (2024) 28:83** 10.1007/s10151-024-02955-x

In this article co-authors’ name was incorrectly published as P. Aeshbacher. The correct name should read as P. Aeschbacher.

